# Post-traumatic stress in adults with 22q11.2 deletion syndrome

**DOI:** 10.1192/bjo.2022.525

**Published:** 2022-07-07

**Authors:** Emma N. M. M. von Scheibler, Thérèse A. M. J. van Amelsvoort, Claudia Vingerhoets, Agnies M. van Eeghen, Erik Boot

**Affiliations:** Advisium, ‘s Heeren Loo Zorggroep, Amersfoort; and Department of Psychiatry and Neuropsychology, MHeNs, Maastricht University, The Netherlands; Department of Psychiatry and Neuropsychology, MHeNs, Maastricht University, The Netherlands; Advisium, ‘s Heeren Loo Zorggroep, Amersfoort; and Emma Children's Hospital, University of Amsterdam, The Netherlands; Advisium, ‘s Heeren Loo Zorggroep, Amersfoort, The Netherlands; Department of Psychiatry and Neuropsychology, MHeNs, Maastricht University, The Netherlands; and The Dalglish Family 22q Clinic, University Health Network, Toronto, Canada

**Keywords:** 22q11.2 deletion syndrome, post-traumatic stress disorder, adults, cognitive–behavioural therapy, eye movement desensitisation reprocessing

## Abstract

22q11.2 deletion syndrome (22q11.2DS) is associated with an elevated genetic risk of several psychiatric disorders. However, the prevalence of post-traumatic stress disorder (PTSD) in individuals with 22q11.2DS has been reported to be only 0.9%; this is lower than that of the general population (3.9%). We explored the occurrence of PTSD and traumatic events in a Dutch cohort of 112 adults with 22q11.2DS, and found PTSD in 8.0%, traumatic events in 20.5% and trauma-focused treatment in 17.9% of patients. Our novel findings suggest that PTSD may be underdiagnosed in individuals with 22q11.2DS. Clinicians and other caregivers should be alert to trauma in this population in order to enable treatment and minimise psychiatric burden.

22q11.2 deletion syndrome (22q11.2DS) is a genetic multisystem disorder with an estimated prevalence of 1 in 2148 live births.^1^ Patients with 22q11.2DS have the highest known genetic risk of schizophrenia, and other psychiatric disorders are collectively even more common.^2^ Counterintuitively, the reported prevalence of post-traumatic stress disorder (PTSD), a psychiatric disorder related to experience of or witnessing a traumatic event, is much lower in 22q11.2DS patients (0.9%) compared with the reported prevalence in the general population (3.9%).^2,3^ Despite advances in treatment of PTSD,^4^ multiple factors including underrecognition of PTSD and experiences of traumatic events could limit implementation of these best practices, contributing to the psychiatric burden in individuals with 22q11.2DS.^5^

We hypothesised a higher prevalence of PTSD in adults with 22q11.2DS compared with the general population.^3^ Therefore, we aimed to explore: (a) the prevalence and potential predictors of PTSD; and (b) the prevalence of potentially traumatic events in 112 adult patients who visited one of the two Dutch specialty clinics.

## Method

As part of ongoing studies on 22q11.2DS, we retrospectively reviewed documentation of direct assessments and available medical records from patients who were 16 years or older at the 22q11.2 out-patient clinic at Maastricht University Medical Centre+ (MUMC) and/or ‘s Heeren Loo.

The authors assert that all procedures contributing to this work comply with the ethical standards of the relevant national and institutional committees on human experimentation and with the Helsinki Declaration of 1975, as revised in 2008. All procedures involving human subjects at MUMC were approved by the medical ethics committee of MUMC (#14-2044, #19-044). A waiver for formal approval of the study at ‘s Heeren Loo was obtained from the medical ethics committee of Amsterdam UMC, The Netherlands (#W20_098). Written informed consent was obtained from all patients and/or legal representatives.

### Sample

We studied data of 112 patients (mean age 32.5 ± 12.4 years, 45% male) with a molecularly confirmed 22q11.2 deletion. Patients were ascertained through referrals from five main sources, from most to least frequent: family medicine, intellectual disability medicine, paediatrics, psychiatry and medical genetics.

### Outcome measures

We recorded information on demographic variables, cognitive functioning (full-scale intelligence quotient; FSIQ) and psychiatric history. A clinical diagnosis of PTSD was the primary outcome measure. We also recorded traumatic events, defined as in DSM-5 subsection A for PTSD (i.e. exposure to actual or threatened death, serious injury or sexual violence according), additional potentially traumatic events affecting daily functioning and any treatment for traumatic events.

### Statistical analysis

We assessed prevalence rates of PTSD and frequencies of patients with a history of traumatic events and/or treatment for trauma in our sample, and calculated 95% confidence intervals using the formula 
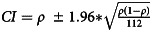
. A logistic regression analysis was used to identify potential predictors of the presence of a clinical diagnosis of PTSD. For this, we considered sex and FSIQ based on previous studies.^6^ All analyses used two-tailed *P*-values with statistical significance defined as *P* < 0.05 using IBM SPSS software (Statistics 25; Inc., Chicago, IL, USA).

## Results

Nine patients (8.0%, 95% CI: 3.0%–13.0%) had a clinical diagnosis of PTSD, of whom two were referred to specialised 22q11 clinics for trauma-related problems and treatment (Table 1). Twenty-three (20.5%) of all patients experienced one or more traumatic events according to DSM-5 criteria, with 12 (10.7%) reporting sexual violence, 11 (9.8%) serious injury (including physical abuse), and four (3.6%) actual or threatened death. Neglect was reported in one patient. An additional 17 patients (15.2%) experienced other potential traumatic events including bullying (*n* = 13, 11.6%), multiple hospital admissions/surgeries (*n* = 4, 3.6%) and out-of-home placement (*n* = 4, 3.6%). Neither sex nor FSIQ was a predictor of a PTSD diagnosis (*P* = 0.58 and *P* = 0.53, respectively).

**Table 1:** Trauma in 112 adults with 22q11.2 deletion syndrome

History of trauma	*N*	%	95% CI
Clinical PTSD diagnosis	9	8.0	3.0−13.0
Traumatic events^a^^,^^b^	23	20.5	13.0−28.0
Sexual violence	12	10.7	5.0−16.4
Serious injury	11	9.8	4.3−15.3
Actual or threatened death	4	3.6	0.2−7.1
Other potential traumatic events^c^	17	15.2	8.6−21.8
Multiple (≥2) traumatic events^d^	14	12.5	6.4−18.6
Treatment for trauma-related conditions			
Treatment for any traumatic event	20	17.9	10.8−25.0
Eye movement desensitisation reprocessing	19	17.0	10.0−24.0
Cognitive–behavioural therapy	2	1.8	0−4.3
Other	2	1.8	0−4.3

PTSD, post-traumatic stress disorder.

a. In one patient with PTSD, traumatic events were not specified.

b. Events meeting DSM-5 criteria for a traumatic event.

c. Events not meeting DSM-5 criteria for a traumatic event.

d. Irrespective of whether DSM-5 criteria were met.

Treatment for trauma was reported in 20 patients (17.9%) and included eye movement desensitisation reprocessing (EMDR; *n* = 19, 17.0%) therapy and cognitive–behavioural therapy (CBT; *n* = 2, 1.8%); one patient received both therapies. Of the nine patients with PTSD, eight were treated with EMDR therapy, including one who received additional CBT. In those with PTSD, the treatment response was noted to be effective in four and minimal to absent in another three patients. For one patient with PTSD, the response to treatment was not reported. Another patient with PTSD did not receive therapy; this was considered not feasible owing to significant neurocognitive decline.

## Discussion

The results of this first explorative study focusing on trauma in adults with 22q11.2DS support our hypothesis of an elevated risk of developing PTSD (8.0%) as compared with the general population (3.9%).^2,3^

There is growing evidence that intellectual disability and borderline intellectual functioning, both often seen in individuals with 22q11.2DS, increase the risk of exposure to traumatic events and the development of PTSD symptoms,^5–7^ with direct negative effects on emotional, behavioural and adaptive functioning.^8^ In addition, life events are more likely to be experienced as traumatic in people with intellectual disabilities, even though DSM-5 criteria are not always met.^5^ However, PTSD and traumatic experiences are often not recognised in these populations.^5,6^ One reason may be that symptoms are overshadowed by or attributed to other psychiatric disorders such as psychotic illness.^6^ Another reason may be that patients and/or their relatives themselves do not recognise trauma-related symptoms^6^ or find it hard to ask for help for trauma-related symptoms. Moreover, professionals may hesitate to pay attention to past traumatic experiences, out of fear of aggravating symptoms and causing a crisis.^6^ Therefore, we presume underrecognition and/or underreporting of PTSD and traumatic experiences in 22q11.2DS. It should also be noted that adults and patients with intellectual disability have been underrepresented in previous 22q11.2DS research.^2^

Recognition of trauma and PTSD is important as it allows for treatment, which may also have the potential to reduce psychosis risk in high-risk populations,^9^ such as people with 22q11.2DS. Although we are not aware of any study reporting on the effectiveness of interventions for trauma in 22q11.2DS, an increasing number of studies show positive effects of EMDR and CBT in individuals with intellectual disability.^10^

### Strengths and limitations

The strengths of this study include the relatively large adult 22q11.2DS sample and the fact that the focus on trauma was not limited to strict DSM-5 criteria for PTSD. There were also several limitations, mostly related to the retrospective nature of the study. For example, data were limited to the available clinical reports. As a consequence, PTSD prevalence may still have been underestimated.^6^ On the other hand, bias towards patients with 22q11.2DS with more severe mental health problems is possible, given that all patients were referred to a 22q11.2DS specialty clinic. Prospective studies are needed to replicate our findings, to further explore the risk of trauma, and to assess the effectiveness of trauma-focused treatments and resilience in 22q11.2DS patients.

## Conclusions

In conclusion, PTSD and traumatic events appear to be more prevalent than previously assumed in adults with 22q11.2DS. Clinicians and other caregivers should thus be alert to PTSD in 22q11.2DS patients in order to minimise the psychiatric burden and reduction in quality of life. Systematic studies in individuals with 22q11.2DS are needed to improve diagnosis, using strategies adjusted to their strengths and weaknesses and including attention to seemingly unimportant life events that may be traumatic, and to evaluate the efficacy of treatments in this population.

## Data Availability

The data that support the findings of this study are available from the corresponding author, E.B., upon reasonable request.
